# Fast synthesis of 1,3-DAG by Lecitase® Ultra-catalyzed esterification in solvent-free system

**DOI:** 10.1002/ejlt.201000507

**Published:** 2011-08

**Authors:** Ning Liu, Yong Wang, Qiangzhong Zhao, Qingli Zhang, Mouming Zhao

**Affiliations:** 1 College of Light Industry and Food Sciences, South China University of TechnologyGuangzhou, China; 2 Department of Food Science and Engineering, Jinan UniversityGuangzhou, China

**Keywords:** 1,3-DAG, Esterification, Lecitase® Ultra, Reaction conditions, Solvent-free system

## Abstract

Lecitase® Ultra, a phospholipase, was explored as an effective biocatalyst for direct esterification of glycerol with oleic acid to produce 1,3-DAG. Experiments were carried out in batch mode, and optimal reaction conditions were evaluated. In comparison with several organic solvent mediums, the solvent-free system was found to be more beneficial for this esterification reaction, which was further studied to investigate the reaction conditions including oleic acid/glycerol mole ratio, temperature, initial water content, enzyme load, and operating time. The results showed that Lecitase® Ultra catalyzed a fast synthesis of 1,3-DAG by direct esterification in a solvent-free medium. Under the optimal reaction conditions, a short reaction time 1.5 h was found to achieve the fatty acid esterification efficiency of 80.3 ± 1.2% and 1,3-DAG content of 54.8 ± 1.6 wt% (lipid layer of reaction mixture mass). The reusability of Lecitase® Ultra was evaluated via recycling the excess glycerol layer in the reaction system. DAG in the upper lipid layer of reaction mixture was purified by molecular distillation and the 1,3-DAG-enriched oil with a purity of about 75 wt% was obtained.

**Practical applications:** The new Lecitase® Ultra catalyzed process for production of 1,3-DAG from glycerol and oleic acid described in this study provides several advantages over conventional methods including short reaction time, the absence of a solvents and a high product yield.

## Introduction

DAGs in different degrees of purity are used as additives or carriers in the food, medicine, and cosmetic industries [1]. Dietary DAGs exhibit antiobesity activity and can prevent postprandial hypertriacylglycerolemia in experimental animals and humans [2–4]. Structurally, DAGs are present in two configurations, namely, 1,3-DAG and 1, 2(2, 3)-DAG. Recently, it is reported that 1,3-DAG oil-enriched diet possesses beneficial effect on humans [5].

Several chemoenzymatic and biotechnological methods are available for the preparation of DAGs [6, 7]. It seems that high yield of 1,3-DAG cannot be obtained by direct chemical methods because the methods lack positional selectivity. The enzymatic synthesis of 1,3-DAG has garnered considerable interests by reason of milder and simpler conditions, higher selectivity, and greener process. In general, DAG can be enzymatically produced by direct esterification, glycerolysis, interesterification, partial hydrolysis, or the combination of partial hydrolysis and esterification [8–12]. As to the direct esterification, since fatty acids and glycerol are immiscible, the common systems are carried out in organic solvents, such as hexane, *t*-butanol, and so forth [13–15]. Nevertheless, enzymatic synthesis of DAG in solvent-free system has attracted more and more attention in recent years [10, 11, 16]. This system could avoid the problems including separation, toxicity, and flammability of organic solvents, thereby, lowering the cost of the final product and permitting the product recovery without further complex purification or evaporation steps.

Lecitase® Ultra (E.C.3.1.1.32) is a phospholipase manufactured and marketed by Novozymes (Copenhagen, Denmark). This commercial preparation is a protein-engineered carboxylic ester hydrolase from the fusion of lipase genes from *Thermomyces lanuginose* and phospholipase genes from *Fusarium oxysporum* [17]. Most of attempts have been performed toward the application of phospholipase activity of this enzyme for degumming of vegetable oils and modifying of phospholipids [18–20]. However, very little effort had been made in exploring applications of its lipase activity in organic synthesis.

In our previous work [21, 22] we have reported the application of Lecitase® Ultra on partial hydrolysis of TAG to produce DAG-enriched oil. It was found that Lecitase® Ultra possessed 1, 3-regiospecficity toward TAG during the partial hydrolysis of soybean oil. And this enzyme had been described to suffer interfacial activation, similarly to lipases [17].

Based on the aspects mentioned above, the main objective of this work was to fast synthesize of 1,3-DAG by direct esterification using Lecitase® Ultra as a catalyst. The enzymatic production of 1,3-DAG by Lecitase® Ultra-catalyzed direct esterification between oleic acid and glycerol in an organic solvent and solvent-free systems was compared. Molecular distillation (MD) was employed to purify 1,3-DAG-enriched oil by removing the excess oleic acid. The new process for fast preparation of 1,3-DAG is more practical for industry due to the lower cost of the enzyme and a short reaction time in a solvent-free system.

## Materials and methods

### Materials

Lecitase® Ultra was purchased from Novozymes. The standards of 1-monoolein, 1, 3-diolein, 1, 2-diolein, and triolein (>99.0%) were purchased from Sigma (St. Louis, MO). Oleic acid and glycerol from Tianjing Chemical Reagent Factory (Tianjing, China) were of analytical grade. All other reagents and solvents used were of analytical or HPLC grade.

### Esterification reaction

The synthesis of 1,3-DAG was performed in a 100 mL round bottom flask, thermostated to the desired operating temperature, and stirred by a magnetic stirrer (200 rpm). The reaction mixture contained the required amount of oleic acid, glycerol, and deionized water. The reaction was started by the addition of a certain amount of Lecitase® Ultra. Then, a certain amount of molecular sieves 4 Å were added for the absorption of water generated during the reaction. After the reactions were completed, each sample was separated into two layers by a centrifuge (at 10 000 × *g* for 10 min at 20°C), the upper lipid layer and the lower glycerol layer.

During this esterification reaction, the effect of oleic acid/glycerol mole ratio, temperature, initial water content, enzyme load, and reaction time on the esterification efficiency of oleic acid and 1,3-DAG content had been studied.

### Reusability of Lecitase® Ultra

In order to evaluate the reusability of Lecitase® Ultra in the esterification reaction, this enzyme was employed for several times via recycling the excess glycerol layer. Each run was performed for 1.5 h, after which the reaction mixtures were centrifuged, the upper lipid layer was collected for analysis and lower glycerol layer including the enzyme was collected for further use. Recycled experiments were carried out by adding fresh oleic acid into glycerol layer containing the enzyme. A certain amount of fresh glycerol was also needed for keeping the constant mole ratio of oleic acid to glycerol. 1,3-DAG content in the upper lipid layer of each run, given as wt %, was analyzed to evaluate the enzyme reusability.

Two types of reuse method of the enzyme were carried out. One process was performed without fresh enzyme addition in recycle runs. The other was done by adding a certain amount of fresh enzyme into each run for the loss of the enzyme activity during the reactions. The fresh enzyme load in the second method was set as 50% of initial enzyme amount, namely, 0.75 wt% of substrates mass.

### Determination of the enzyme activity

The determination procedure of lipase activity of Lecitase® Ultra was modified according to the method described by Mishra et al. [23]. The enzyme activity was determined by titration using glycerol tributyrate (tributyrin) as the substrate in a pH-stat mode at 30°C in a 0.005 mol/L Tris–HCl buffer (pH 7.5) containing 0.025 mol/L CaCl_2_. One unit of Lecitase® Ultra was defined as the amount of enzyme which released 1 µmol of titratable free butyric acid per minute under the described conditions. The activity of the commercial Lecitase® Ultra using tributyrin was found to be 3326 units/mL.

### Determination of esterification efficiency

The upper lipid layer from the reaction mixture after centrifugation was titrated with 0.05 mol/L KOH solution to determine the amount of unconverted fatty acid in terms of acid value (AV). The esterification efficiency was defined as the consumed oleic acid amount during the reaction.

### Acylglycerols compositions analyzed by normal-phase HPLC (NP-HPLC)

The compositions of the upper lipid layer were determined by a NP-HPLC system (Waters, MA, USA) equipped with a dual *λ* absorbance detector. The chromatographic separation was carried out with a Grace silica column (5 µm, 250 mm × 4.6 mm). Gradient elution was achieved by mobile phase A (*n*-hexane/isopropanol = 99:1 v/v) and B (isopropanol). The gradient was operated as follows: 0–35 min: 100% A; 35–55 min: 80% A and 20% B; 55–65 min: 100% A, and the flow rate was fixed at 1.0 mL/min. The UV detection wavelength was set at 210 nm. All samples were filtered through 0.45 µm nylon membrane filter prior to HPLC analysis. Injection volume of 20 µL was kept constant for all samples during analysis. The compositions of acylglycerols were calculated as weight percentage of the samples analyzed using calibration curves of standards.

### Molecular distillation

The upper lipid layer of the reaction mixtures was composed of excess oleic acid, MAG, 1,3-DAG, 1,2-DAG, and TAG. The 1,3-DAG was purified by a MD-80 equipment (Handway Technology Foshan Co. Ltd., Foshan, China). The areas of evaporator and condenser were 0.066 and 0.05 m^2^, respectively, and the space between them was 30 mm. Oleic acid and MAG were removed from the mixture by MD at an evaporator temperature of 180°C. Other conditions for MD were as followings: evaporator vacuum, 0.5–1.0 Pa; roller speed, 300 rpm; condenser temperature, 40°C; feed rate, 0.5 L/h; and feed temperature, 80°C.

## Results and discussion

### Effect of solvents on the esterification reaction

In general, organic solvent characteristics not only affect the mass transfer in the reaction system, but also impact the conformation and activity of the enzyme involved. The physicochemical effect on the enzyme molecules differs depending upon the kinds of organic solvents and the enzymes used. In this work, several organic solvents with different log *p*-values (*p*-the partitioning coefficient between 1-octanol and water) were applied to the Lecitase® Ultra-catalyzed esterification reaction mediums. These solvents were selected with a wide range log *p*-values from −0.23 to 4.5. Table 1 illustrates the effect of different organic solvents on the esterification efficiency. It was clear that both esterification efficiency of oleic acid and 1,3-DAG content were dependent on the solvents to a great extent. The solvents with log *p* > 3.5 were positive for Lecitase® Ultra catalysis, while those with log *p* < 3.5 were nearly negative. Obviously, the higher log *p*-values the organic solvents (*n*-hexane, *n*-heptane, and isooctane) possessed, the greater esterification efficiency of oleic acid was achieved. This tendency in reactivity conformed to the general rule for solvent suitability in supporting microaqueous biocatalysis. Kuo and Parkin [24] reported part of the basis for this phenomenon resided with the water-sorption ability of the more polar solvent (with low log *p*-values) and the attendant competition with biocatalyst for water in the reaction mixture.

**Table 1:** Effect of organic solvents on esterification synthesis of 1,3-DAG

Solvent	Log *p*	Esterification efficiency (%)	1,3-DAG content (wt%)
Acetone	−0.23	4.8 ± 0.2	Trace
Isopropanol	0.28	5.3 ± 0.1	Trace
*t*-Butanol	0.80	9.9 ± 0.3	Trace
Chloroform	2.0	8.1 ± 0.5	Trace
Toluene	2.5	9.2 ± 0.4	Trace
Tetrachlor-omethane	3.0	11.7 ± 0.6	Trace
*n*-Hexane	3.5	74.5 ± 1.2	50.4 ± 0.2
*n*-Heptane	4.0	72.8 ± 1.9	51.8 ± 1.0
Isooctane	4.5	72.0 ± 0.6	53.4 ± 1.4
Solvent-free		79.3 ± 0.8	55.5 ± 0.5

Reaction conditions: temperature, 45°C; 12 h; mole ratio oleic acid/glycerol 1:7.5; solvent amount, solvent/substrates 1:1 w/w; enzyme load, 2 wt% of substrates; initial water content, 4 wt% of substrates; 4 Å molecular sieves, 10 wt% of substrates. Data are mean values ± SD (*n* = 3).

Nevertheless, some other researchers [25, 26] suggested that the miscibility of the reaction media was one of the most essential factors influencing the enzymatic reaction. Those amphiphilic solvents, such as isopropanol and *t*-butanol with low log *p*-values, were tertiary alcohols with both hydrophilic and hydrophobic characteristics which could improve the miscibility of fatty acids and glycerol. This amphiphilic characteristic might also improve the mass transfer, which resulted in accelerating the reaction rate. However, in our study, it was observed that these amphiphilic solvents inhibited the esterification reaction between oleic acid and glycerol catalyzed by Lecitase® Ultra. This explanation might be that Lecitase® Ultra was a free enzyme solution, and the amphiphilic solvents would strip the water molecules around the enzyme molecules. In other words, esterification activity of Lecitase® Ultra was probably decreased in relatively polar solvent systems due to strip of moisture from the enzyme.

The most interesting finding was observed in the solvent-free system, which had the highest fatty acid esterification effiency (79.3 ± 0.8%) and 1,3-DAG content (55.5 ± 0.5 wt%). It was known that the lipase-catalyzed esterification reaction of fatty acids and glycerol was induced on the interface. In a solvent-free system, therefore, there might be more chances for the enzyme molecules to get in touch with the substrates on the interface, and they were allowed to change the conformations more beneficially to reveal catalysis activity. As a result, the solvent-free system was selected as the Lecitase® Ultra-catalyzed esterification reaction medium in this study.

### Effect of substrates mole ratio

**Figure 1:**
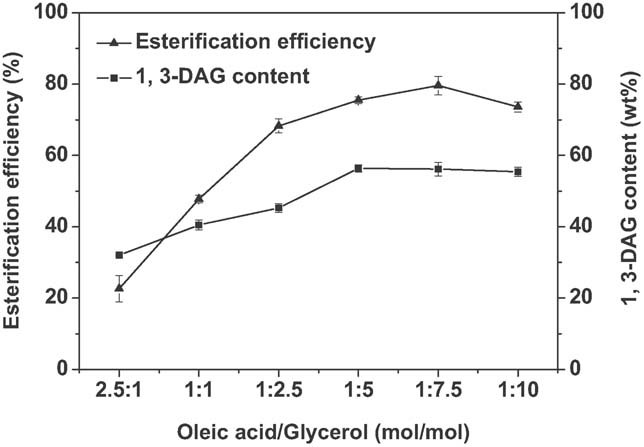
Effect of oleic acid/glycerol mole ratio on the esterification synthesis of 1,3-DAG. Reaction conditions: temperature, 45°C; 6 h; enzyme load, 2 wt% of substrates; initial water content, 4 wt% of substrates; 4 Å molecular sieves, 10 wt% of substrates. Data are mean values of duplicate.

The esterification reactions were carried out at different mole ratios of oleic acid to glycerol by Lecitase® Ultra-catalyzed at 45°C in solvent-free system and the results are shown in Fig. 1. It showed that the esterification efficiency of oleic acid increased with increasing the mole ratio of glycerol. The highest esterification efficiency was achieved when the mole ratio was 1:7.5. Yesiloglu and Kilic [13] reported that the optimum mole ratio of oleic acid to glycerol was 1:10 catalyzed by *Candida rugosa* lipase and 1:25 catalyzed by porcine pancreas lipase because of the excess glycerol absorbed water formed during the esterification, which shifted the equilibrium toward the esterification and higher extent of esterification efficiency of oleic acid was obtained. On the other hand, as shown in Fig. 1, the optimum mole ratio of oleic acid/glycerol for the highest 1,3-DAG content (56.4 wt%) was 1:5. Based on the standpoint of the 1,3-DAG content, the oleic acid/glycerol mole ratio for 1,3-DAG production was selected as 1:5 for this synthesis.

### Effect of reaction temperature

**Figure 2:**
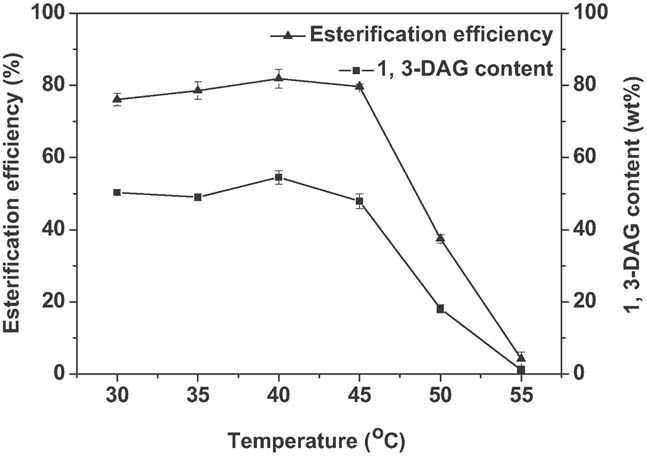
Effect of reaction temperature on the esterification synthesis of 1,3-DAG. Reaction conditions: 6 h; mole ratio oleic acid/glycerol, 1:5; enzyme load, 2 wt% of substrates; initial water content, 4 wt% of substrates; 4 Å molecular sieves, 10 wt% of substrates. Data are mean values of duplicate.

The effect of reaction temperature from 30 to 55°C on esterification reaction in solvent-free system is shown in Fig. 2. From 30 to 40°C, the esterification efficiency and 1,3-DAG content increased with the increase of reaction temperature simultaneously. Both esterification efficiency and 1,3-DAG content reached the maximums (81.8 ± 2.5% and 54.5 ± 1.8 wt%, respectively), at 40°C. Lecitase® Ultra displayed good activity below 45°C, however, the reaction temperature higher than 45°C would cause reversible and irreversible denaturation of the enzyme which was negative to the reaction. Herein, reaction temperature 40°C was selected as the optimal catalysis temperature.

The temperature has great effect on the catalyzing activity of the enzyme molecules as well as the thermodynamic equilibrium of a reaction. Every kind of enzyme has its optimal temperature. Mishra et al. [23] reported that Lecitase® Ultra was stable up to 50°C and quickly lost its activity above 60°C in 0.05 mol/L Tris–HCl buffer containing 0.025 mol/L CaCl_2_.

### Effect of initial water content

**Figure 3:**
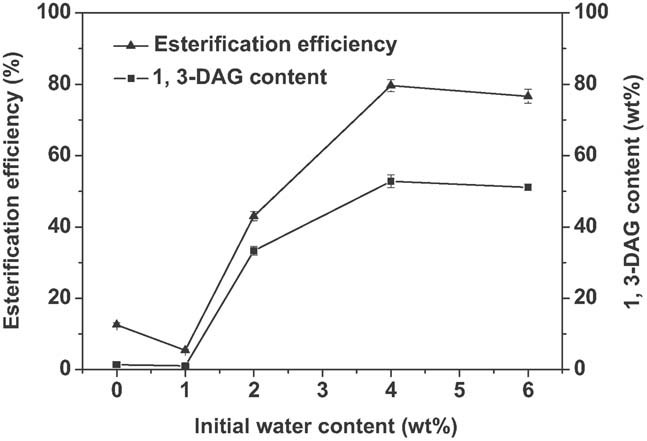
Effect of initial water content on the esterification synthesis of 1,3-DAG. Reaction conditions: temperature, 45°C; 6 h; mole ratio oleic acid/glycerol, 1:5; enzyme load, 2 wt% of substrates. Data are mean values of duplicate.

In microaqueous medium a certain amount of water in the enzyme vicinity is necessary for the enzyme to keep active and the optimal hydration level varies from enzyme species [27]. Hence, the initial water is required for the enzyme-catalyzed reaction. On the other hand, water is a product of esterification reaction. An excess water remaining in the reaction mixture shift the equation toward the reversed reaction, namely hydrolysis. So, some procedures are used for water removal in esterification reaction, one of which is the addition of molecular sieve. In our research, however, the effect of molecular sieve 4 Å on the esterification efficiency was found not to be significant (data not shown). Monot et al. [27] had also reported the removal of water had little effect on the yield of esterification. Therefore, in the subsequent experiments, molecular sieve 4 Å was not added into the reaction mixture. The effect of the initial water content on the reaction is demonstrated in Fig. 3. When Lecitase® Ultra was in a system with absence of the initial water, esterification reaction did not nearly occurred and there was little 1,3-DAG produced in this work. With an initial water content of 4 wt% in the substrates mixture, the maximum esterification efficiency of 79.6 ± 1.7% and 1,3-DAG content of 52.8 ± 1.6 wt% were achieved, respectively. The optimal initial water is essential to maintain the conformation of Lecitase® Ultra to display the high activity to the reaction.

### Effect of enzyme load

**Figure 4:**
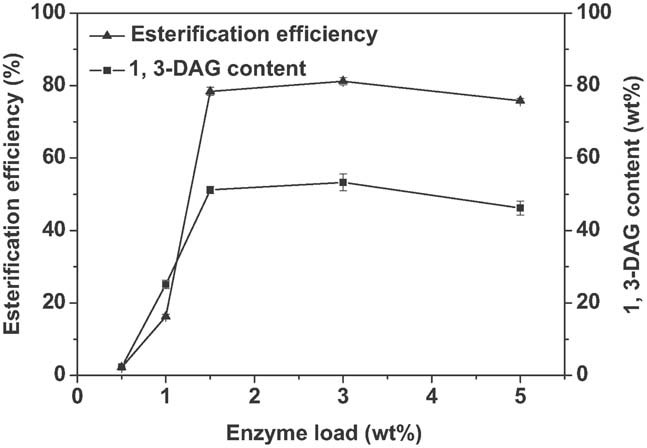
Effect of enzyme load on the esterification synthesis of 1,3-DAG. Reaction conditions: temperature, 45°C; 6 h; mole ratio oleic acid/glycerol, 1:5; initial water content, 4 wt% of substrates. Data are mean values of duplicate.

As presented in Fig. 4, when the enzyme load was at 1.5 wt% (substrates mass), the esterification efficiency of 78.4% and the 1,3-DAG content of 51.2 ± 0.9 wt% were obtained. However, an enzyme load of over 3.0 wt% (substrates mass) showed no advantages on the esterification efficiency and the 1,3-DAG content. The esterification efficiency and the 1,3-DAG content increased rapidly at low value of enzyme load, but decreased slightly with the further increase of the enzyme dosage. Enzyme agglomeration and diffusion problems at a high dosage caused the decline of reaction efficiency [15]. Therefore, 1.5 wt% (substrates mass) was selected as the optimum enzyme load in this work.

### Effect of reaction time

**Figure 5:**
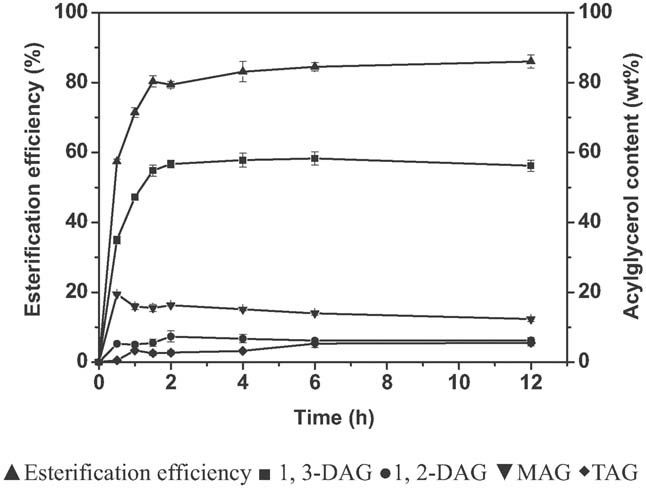
Effect of reaction time on the esterification synthesis of 1,3-DAG. Reaction conditions: temperature, 40°C; mole ratio oleic acid/glycerol, 1:5; enzyme load, 1.5 wt% of substrates; initial water content, 4 wt% of substrates. Data are mean values of duplicate.

Figure 5 shows the effect of reaction time on the esterification efficiency and the 1,3-DAG content. The esterification efficiency of 71.4 ± 1.4% was obtained at 1 h. The 1,3-DAG content in the upper layer reached the maximum (54.8 ± 1.6 wt%) at 1.5 h and increased slightly with the increase of reaction time. The contents of 1,2-DAG and TAG were nearly constant at a low level during the reaction. The reason might be that Lecitase® Ultra possessed a 1, 3-regiospecficity and oleic acid was esterified with glycerol at *sn*-1 and *sn*-3 position to form 1,3-DAG rather than 1,2-DAG, which was complied with our former study [21]. As a result, 1.5 h reaction was adequate to produce 1,3-DAG when Lecitase® Ultra was used as the catalyst at a suitable load. The high efficiency was attributed to the high activity of the enzyme to oleic acid under the optimized reaction conditions above. The 1,3-DAG content obtained under Lecitase® Ultra-catalyzed esterification was approximate to those obtained in some lipase-catalyzed systems. Rosu et al. [10] reported 1,3-DAG concentration of about 61% from the esterification of oleic acid with glycerol in solvent free system. Yesiloglu and Kilic [13] reported total DAG concentration of 39.3% from the esterification of oleic acid with glycerol in *n*-hexane system catalyzed by *C. rugosa* lipase. The difference of DAG yield between the above two studies and that obtained in our study was mainly ascribed to the different enzyme used, different solvent employed, and reaction time. Watanabe et al. [28] reported the maximum yield of 84% and a purity of 90% of 1,3-DAG was obtained using the lipase Lipozyme RM IM as a catalyst in the esterification reaction. Different substrates and enzymes used led to the different results. Fatty acids employed by Watanabe et al. were the uncrystallized liquid fractions of hydrolyzates from soybean oil and rapeseed oil, which were mainly linoleic acid and oleic acid. However, in the present study, oleic acid (analytical grade) was used as the substrate. Rosu et al. [10] reported the yield of 1,3-DAG from oleic acid was lower than that from linoleic acid by esterification in a solvent-free system at 25°C. Furthermore, as generated from different kind of microorganism, Lecitase® Ultra used in this study should possess different enzyme activity and 1, 3-regiospecficity over the lipase Lipozyme RM IM, resulting in a relatively lower 1,3-DAG yield in the present study.

### Reusability of Lecitase® Ultra

**Figure 6:**
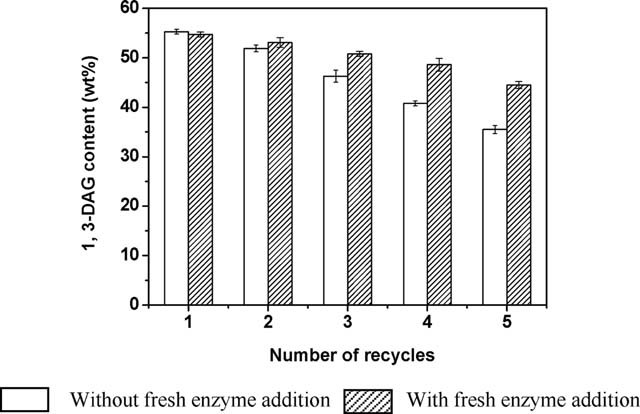
The reusability of Lecitase® Ultra during the esterification synthesis of 1,3-DAG. Reaction conditions: temperature, 40°C; mole ratio oleic acid/glycerol, 1:5; initial water content, 4 wt% of substrates; enzyme load, 1.5 wt% of substrates (fresh enzyme load, 0.75 wt% of substrates). Data are mean values of duplicate.

The result of recycling the glycerol layer is demonstrated in Fig. 6. As shown, without addition of fresh enzyme, the 1,3-DAG content in the lipid layer declined from 55.3 ± 0.5 to 32.6 ± 0.8 wt% after five recycle runs. Whereas, by adding 0.75 wt% (substrates mass) enzyme into each run, after five runs, the 1,3-DAG content in the lipid layer was 47.6 ± 0.6 wt% which only decreased approximately 7 wt% as compared with that in the first run. Therefore, the cost of enzyme was reduced significantly in reactions for several times. The recycle of the excess glycerol layer with addition half amount of fresh enzyme in esterification reaction would save half of the enzyme load which was economically attractive when application in the industry.

### Molecular distillation

The final product of 1,3-DAG-enriched oil was obtained by MD. MD is generally accepted as a safety method to separate and to purify thermally unstable compounds and substances with low vapor pressures and high molecular weights, without significant thermal decomposition. In the present work, MD in laboratory scale was performed. The residue oleic acid and MAG generated during the reaction was removed by MD. Since the contents of 1,2-DAG and TAG were at small amounts in the mixture, the residue from MD was defined as 1,3-DAG-enriched oil. Actually, MD has been widely used in lipid areas [29, 30]. As to some enzymatic reactions, the purity of target product is not high enough so that it is necessary to resort to some purification processes. Fregolente et al. [31] had used MD as the purification method for the production of monoglyceride and diglyceride combined with the lipase-catalyzed glycerolysis of soybean oil. Xu et al. [32] studied the lipase-catalyzed acidolysis between rapeseed oil and capric acid for production of structural lipid, after which the MD parameters for purification and deodorization of product was optimized. Yang et al. [12] also employed MD to purify the DAG produced by glycerolysis from butterfat.

In this study, the yield of the product (heavy phase) from MD was 53.1 ± 0.4%. It seemed to be low in some extent. For a batch MD, there was some residual feed stuff in the machine which decreased the yield of the final products. However, the effect of the residual on the yield would be minimized by a continuous MD if the enough feed stuff was obtained. Interestingly, the light phase from MD which mainly containing the residue oleic acid could be recycled for esterification reaction again to improve the utilization of the raw materials. Furthermore, in the present work, the 1,3-DAG content of the final oil product was about 75 wt%. It was a potential application in functional oils. Fregolente et al. [31] studied the lipase-catalyzed glycerolysis of soybean oil for production of DAG as well as the MD process for purifying the product. They reported a DAG-enriched oil with unhydrolyzed TG and residual MG (29.8% of TG, 53.2% of DG, and 15.6% of MG), which was suitable to replace TG oil in the human diet.

## Conclusions

Lecitase® Ultra was successfully used as a catalyst for synthesis of 1,3-DAG by a direct esterification in a solvent-free system with a high esterification efficiency and a yield of 1,3-DAG. The optimal conditions selected for the esterification were as follows: oleic acid/glycerol mole ratio 1:5, temperature 40°C, initial water content 4 wt% (substrates mass), enzyme load 1.5 wt% (substrates mass), and reaction time 1.5 h. The excess glycerol layer in esterification reaction enabled Lecitase® Ultra to be recycled for 1,3-DAG production. After MD, a purity of about 75 wt% 1,3-DAG-enriched oil was obtained. The results obtained in the present study should be helpful for researchers who are interested in exploring the application of Lecitase® Ultra in lipid areas.

## Abbreviation

MD: molecular distillation
